# Health-related quality of life outcomes from KEYNOTE-412: chemoradiotherapy with or without pembrolizumab in participants with head and neck squamous cell carcinoma

**DOI:** 10.3389/fonc.2025.1645509

**Published:** 2025-10-06

**Authors:** J.-P. Machiels, Y. Tao, L. Licitra, B. Burtness, M. Tahara, D. Rischin, G. V. Alves, I. P. F. Lima, B. G. M. Hughes, Y. Pointreau, S. Aksoy, S. Laban, R. Greil, M. Burian, M. Hetnal, J.-P. Delord, R. Mesia, M. Taberna, J. Waldron, C. Simon, V. Gregoire, K. Harrington, C. M. Black, J. M. Norquist, A. Wang, B. Gumuscu, B. Bidadi, L. L. Siu

**Affiliations:** ^1^ Department of Medical Oncology, Cliniques Universitaires Saint-Luc and Institut de Recherche Expérimentale et Clinique (Pole MIRO), UCLouvain, Brussels, Belgium; ^2^ Department of Radiation Oncology, Gustave Roussy, Villejuif, France; ^3^ Head and Neck Cancer Medical Oncology Department, Fondazione IRCCS Istituto Nazionale dei Tumori and University of Milan, Milan, Italy; ^4^ Yale University School of Medicine and Yale Cancer Center, New Haven, CT, United States; ^5^ Division of Head and Neck Medical Oncology, National Cancer Center Hospital East, Kashiwa, Japan; ^6^ Medical Oncology, Peter MacCallum Cancer Centre and the University of Melbourne, Melbourne, VIC, Australia; ^7^ Medical Oncology, Centro Integrado de Pesquisa em Oncología, Hospital Nossa Senhora da Conceição, Porto Alegre, Brazil; ^8^ Department of Oncology, CRIO Centro Regional Integrado de Oncología, Fortaleza, Brazil; ^9^ Royal Brisbane and Women’s Hospital and University of Queensland, Brisbane, QLD, Australia; ^10^ Department of Radiation Oncology, Centre Jean Bernard Institut Inter-régionaL de Cancérologie, Centre de Cancérologie de la Sarthe, Le Mans, France; ^11^ Department of Medical Oncology, Hacettepe University Cancer Institute, Ankara, Türkiye; ^12^ Head & Neck Cancer Center of the Comprehensive Cancer Center Ulm, Ulm University Medical Center, Ulm, Germany; ^13^ Department of Internal Medicine, Paracelsus Medical University, Salzburg Cancer Research Institute, and Cancer Cluster Salzburg, Salzburg, Austria; ^14^ Krankenhaus der Barmherzigen Schwestern Linz, Linz, Austria; ^15^ Andrzej Frycz Modrzewski Krakow University, Amethyst Radiotherapy Center, Rydygier Hospital, Krakow, Poland; ^16^ European Cancer Organisation, Brussels, Belgium; ^17^ Medical Oncology Department, Catalan Institute of Oncology - Badalona, B-ARGO group, CARE program, IGTP, Badalona, Spain; ^18^ Savana, Madrid, Spain; ^19^ Department of Radiation Oncology, Princess Margaret Cancer Centre, Toronto, ON, Canada; ^20^ Department of Otolaryngology-Head and Neck Surgery, University Medical Center Vaud (CHUV) - Lausanne University Hospital, Lausanne, Switzerland; ^21^ Radiation Oncology Department, Léon Bérard Cancer Center, Lyon, France; ^22^ The Institute of Cancer Research, London, United Kingdom; ^23^ Merck & Co., Inc., Rahway, NJ, United States; ^24^ Division of Medical Oncology and Hematology, Princess Margaret Cancer Centre, University of Toronto, Toronto, ON, Canada

**Keywords:** head and neck cancer, health-related quality of life, immunotherapy, patient reported outcomes, pembrolizumab, chemoradiotherapy

## Abstract

**Background:**

The health-related quality of life (HRQoL) of patients with locally advanced head and neck squamous cell carcinoma (LA HNSCC) is impacted by both disease- and treatment-related factors. Treatments that preserve and maximize HRQoL in this setting represent a substantial unmet need.

**Methods:**

KEYNOTE-412 (NCT03040999) was a randomized, double-blind, placebo-controlled phase 3 study of pembrolizumab plus chemoradiotherapy (CRT) versus placebo plus CRT for maintenance therapy in participants with treatment-naïve LA HNSCC. Patient-reported outcomes (PROs) assessed using the European Organisation for Research and Treatment of Cancer Quality of Life Questionnaire Core 30 (EORTC QLQ-C30) and EORTC QLQ Head and Neck 35 (H&N35) were pre-specified secondary endpoints and administered at baseline and throughout the study. Least squares mean (LSM) change from baseline was assessed using a constrained longitudinal data analysis model. No formal statistical significance testing was performed.

**Results:**

The PRO analysis population included 395 participants randomized to receive pembrolizumab plus CRT and 397 to receive placebo plus CRT. Completion rates for all assessed PROs were >95% at baseline and >66% at week 45. LSM change from baseline to week 45 was similar between groups across EORTC QLQ-C30 and QLQ-H&N35 subscale scores. There were no notable differences in empirical mean change or the proportion of participants with improvement, stability, or deterioration from baseline to week 45 between treatment groups.

**Conclusion:**

The addition of pembrolizumab to CRT did not meaningfully impact HRQoL in participants with LA HNSCC.

## Introduction

Head and neck squamous cell carcinomas (HNSCCs) often arise in the oral cavity, pharynx, and larynx (1). HNSCCs can cause significant morbidity because these sites are central to basic physiological (breathing and swallowing), sensory (taste and smell), and personal characteristics (appearance and speech) (2). Treatments with curative intent for locally advanced (LA) HNSCCs, including surgery and concurrent chemoradiotherapy (CRT), have improved disease control, but also risk further impairing functional and cosmetic outcomes and profoundly impact patient quality of life (QoL) (2–6).

Health-related QoL (HRQoL) has emerged as a critical end point in clinical studies of patients with head and neck cancers (6, 7), offering insights into the impact of cancer and its treatment from the patient’s perspective. By directly assessing the patient’s perspective of treatment, patient-reported outcomes (PROs) can provide a more complete picture of treatment outcomes in clinical studies (8, 9). Difficulty with swallowing, difficulty with speech, and pain have been identified as the most clinically meaningful symptoms for patients with HNSCC (10), highlighting the unmet need for treatment options that preserve and maximize function and HRQoL.

In the phase 3 KEYNOTE-412 study of participants with LA HNSCC, pembrolizumab plus CRT followed by pembrolizumab maintenance therapy did not significantly improve event-free survival (EFS) compared with placebo plus CRT (hazard ratio [95% confidence interval]: 0.83 [0.68–1.03]; *P* = 0.0429), and no new safety signals were observed (11). This paper reports the pre-specified secondary and exploratory PRO end points of KEYNOTE-412 and evaluates the impact of adding pembrolizumab to CRT as maintenance therapy on HRQoL.

## Methods

KEYNOTE-412 (NCT03040999) was a double-blind, multicenter, randomized, placebo-controlled phase 3 study designed to evaluate the efficacy and safety of pembrolizumab plus CRT compared with placebo plus CRT as maintenance therapy in participants with treatment-naïve LA HNSCC.

The study was conducted in accordance with the principles of Good Clinical Practice and was approved by the appropriate institutional review boards and regulatory agencies. All participants provided written informed consent.

### Study population

Participants were eligible for enrollment if they were at least 18 years old and had a pathologically proven new diagnosis of LA HNSCC (either T3-T4 [N0-N3] M0 or any N2-3 [T1-T4] M0 larynx/hypopharynx/oral cavity/p16-negative oropharynx cancers, or either T4 [N0-N3] M0 or N3 [T1-T4] M0 p16-positive oropharynx cancer). Other key eligibility criteria included no prior treatment for the HNSCC under investigation, an Eastern Cooperative Oncology Group performance status (ECOG PS) score of 0–1, a tumor burden that was evaluable by Response Evaluation Criteria in Solid Tumors (RECIST) v1.1, and being a candidate for definitive high-dose cisplatin-based CRT.

### Study design

Eligible participants were randomly assigned 1:1 to receive intravenous pembrolizumab 200 mg or placebo every 3 weeks plus CRT for either 17 cycles of pembrolizumab/placebo or until disease progression, intolerable toxicity, or physician or participant decision to withdraw from the study. CRT included intravenous cisplatin 100 mg/m^2^ every 3 weeks plus accelerated fractionation radiotherapy (70 Gy, 6 fractions/week for 6 weeks [5 fractions in the final week]; 35 fractions in total) or standard fractionation radiotherapy (70 Gy, 5 fractions/week for 7 weeks; 35 fractions in total). Pembrolizumab and placebo were first administered as a priming dose 1 week prior to CRT, followed by 2 doses during CRT, and 14 doses as maintenance therapy after CRT.

Randomization was stratified by radiotherapy regimen (accelerated vs standard fractionation), p16 status (p16-positive oropharyngeal tumors vs p16-negative oropharyngeal or laryngeal/hypopharyngeal/oral cavity tumors), and tumor stage (III vs IV).

### Study outcomes

The primary study end point was event-free survival (defined as the time from randomization to radiographically or pathologically confirmed progressive disease, surgery, or death due to any cause, whichever occurred first), which has been reported elsewhere (11).

Pre-specified secondary end points included change from baseline in European Organisation for Research and Treatment of Cancer Quality of Life Questionnaire Core 30 (EORTC QLQ-C30) global health status/quality of life (GHS/QoL) score, EORTC QLQ-C30 physical functioning score, EORTC QLQ-Head and Neck 35 (H&N35) swallowing symptom score, EORTC QLQ-H&N35 speech symptom score, and EORTC QLQ-H&N35 pain symptom score. HRQoL utilities were also assessed as an exploratory end point using the EuroQoL five dimensions visual analog scale (EQ-5D VAS).

### Study assessments and procedures

EORTC questionnaires are widely used to assess the QoL of patients with cancer. The QLQ-C30 contains 5 functional scales (physical, role, cognitive, emotional, and social), 3 symptom scales (fatigue, nausea and vomiting, and pain), 6 single-symptom items (dyspnea, sleep disturbance, appetite loss, constipation, diarrhea, and financial impact), and a GHS/QoL dimension (12). The QLQ-H&N35 contains 7 multi-item scales (pain in the mouth, problems with swallowing, senses, speech, social eating, social contact, and sexuality) and 11 single-item scales (problems with teeth, mouth opening, dry mouth, sticky saliva, coughing, feeling ill, use of analgesics, use of nutritional supplements, use of feeding tube, weight gain, and weight loss) (13). The EORTC QLQ-C30 and QLQ-H&N35 are scored from 0–100, with higher scores for functioning scales and GHS indicating better functioning and higher scores for symptom and single-item scales indicating worsening symptoms. These instruments have been psychometrically and clinically validated in patients with head and neck cancers, and a ≥10-point difference on the QLQ-C30 and QLQ-H&N35 scales (either from baseline or between treatment groups) is considered clinically relevant (14, 15).

The EQ-5D is a standardized instrument that has been used extensively in oncology studies to measure health outcomes. The health state dimensions in the EQ-5D include mobility, self-care, usual activities, pain/discomfort, and anxiety/depression, as well as a visual analog scale graded from 0–100 for general state of health at the time of the assessment (with higher scores indicating higher HRQoL).

PRO questionnaires were administered electronically at the study site at baseline, week 6, and week 9, then every 12 weeks during the maintenance phase. PROs were also completed at treatment discontinuation and 30 days after the last dose of treatment at the safety follow-up visit, then every 3 months during year 2 and once a year until year 5. PRO questionnaires were completed prior to other study procedures, including the administration of study treatment; the EQ-5D was administered first, followed by the EORTC QLQ-C30, then the EORTC QLQ-H&N35.

### Statistical analysis

PRO analyses were assessed in all randomly assigned participants who completed at least one PRO and received at least one dose of study treatment. PRO analyses were conducted at prespecified questionnaire completion rates of at least 60% and compliance rates of at least 80%. Completion was defined as the proportion of participants who completed at least one questionnaire at each time point among those in the PRO analysis population. Compliance was defined as the proportion of participants who completed at least one questionnaire at each time point among those who were expected to complete the instruments at that time point, excluding those missing by design (such as death, discontinuation, and translations not available).

A constrained longitudinal data analysis model with PRO scores as the response variable and treatment-by-time interaction and stratification factors as covariates was used to estimate the LSM change from baseline and between-group difference in EORTC QLQ-C30 and QLQ-H&N35 PROs. EORTC QLQ-C30 and QLQ-H&N35 PROs were also assessed by rates of overall improvement (≥10-point increase [in the positive direction] from baseline at any time, confirmed by a ≥10-point increase from baseline at the next consecutive visit), stability (when criteria for improvement are not met, a change in score of <10 points from baseline at any time that is confirmed at the next consecutive visit), and deterioration (≥10-point decrease from baseline at any time when none of the criteria for improvement or stability are met).

## Results

Overall, 804 participants were randomly assigned to receive pembrolizumab plus CRT (n=402) or placebo plus CRT (n=402). Baseline characteristics were well balanced between treatment groups (**Supplementary Table S1**). The most common tumor site for both groups was oropharynx (n=200, pembrolizumab plus CRT; n=204, placebo plus CRT) followed by larynx (n=92, pembrolizumab plus CRT; n=86, placebo plus CRT), hypopharynx (n=71, pembrolizumab plus CRT; n=73, placebo plus CRT), and oral cavity (n=39, pembrolizumab plus CRT; n=39, placebo plus CRT). At baseline, most participants were stage T3-T4b (n=335, pembrolizumab plus CRT; n=347, placebo plus CRT) and overall stage IVa-IVb (n=262, pembrolizumab plus CRT; n=265, placebo plus CRT) (**Supplementary Table S1**). Most participants in both groups had a PD-L1 combined positive score of at least 1 (CPS ≥1; n=339, pembrolizumab plus CRT; n=346, placebo plus CRT). The median time from randomization to the database cutoff date (31 May 2022) was 47.7 months (IQR, 42.1–52.3). The PRO population included 792 participants (n=395 in the pembrolizumab plus CRT group and n=397 in the placebo plus CRT group).

Completion and compliance rates were >95% at baseline for all PRO questionnaires across treatment groups (**Supplementary Table S2**). At week 45, completion rates for the EORTC QLQ-C30 were 67.8% in the pembrolizumab plus CRT group and 67.0% in the placebo plus CRT group, and compliance rates were 97.1% and 97.4%, respectively. For the EORTC QLQ-H&N35, completion rates were 67.8% in the pembrolizumab plus CRT group and 66.9% in the placebo plus CRT group, and compliance rates were 97.1% and 97.4%, respectively. For the EQ-5D, completion rates were 67.8% in the pembrolizumab plus CRT group and 67.0% in the placebo plus CRT group, and compliance rates were 90.5% and 93.3%, respectively.

**Figure 1:**
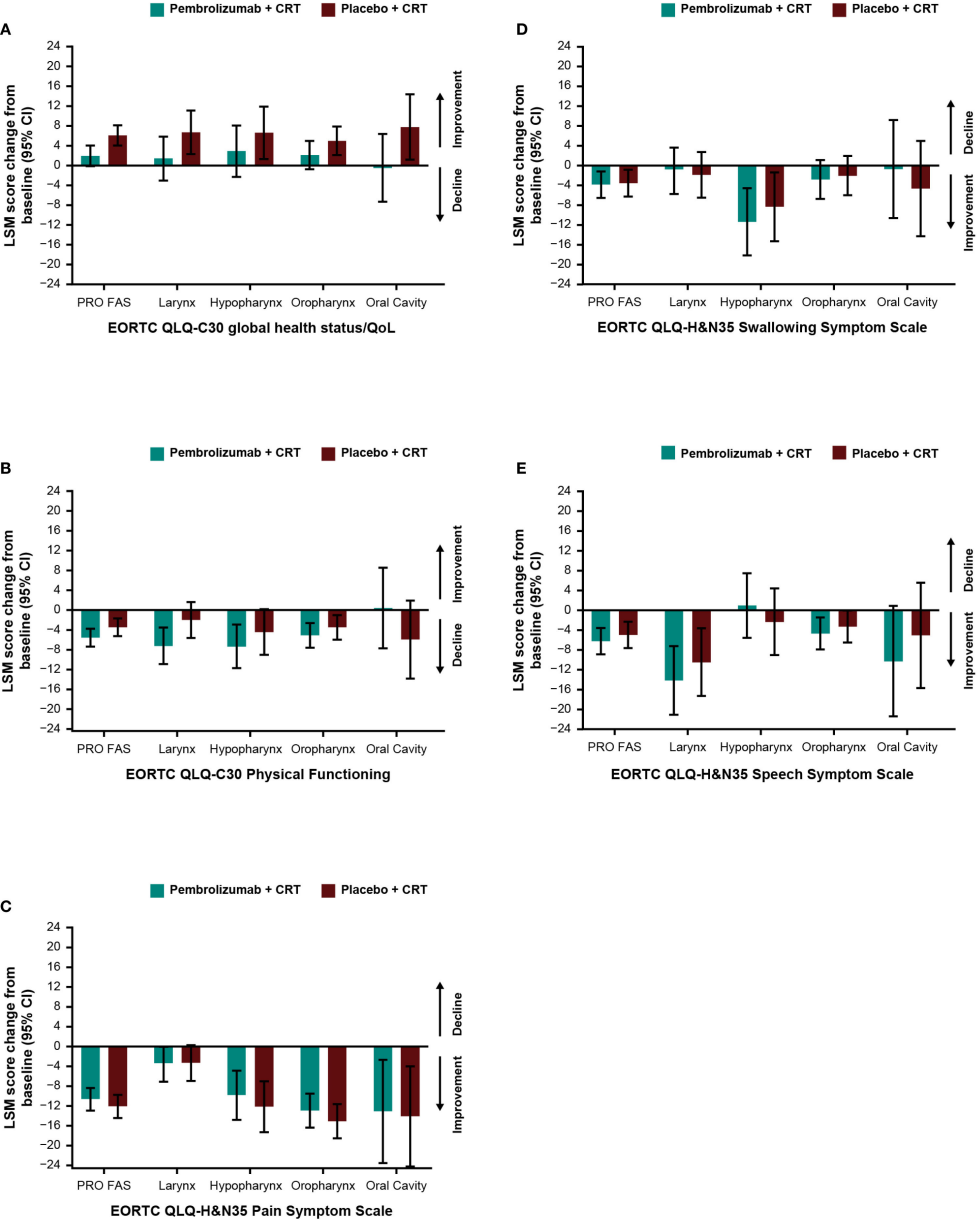
Difference in least squares mean from baseline to week 45 by primary tumor site location in **(A)** EORTC QLQ-C30 GHS/QoL, **(B)** EORTC QLQ-C30 PF, **(C)** EORTC QLQ-H&N35 pain, **(D)** EORTC QLQ-H&N35 swallowing, and **(E)** EORTC QLQ-H&N35 speech scores. C30, Core 30; CI, confidence interval; CRT, chemoradiotherapy; EORTC QLQ, European Organisation for Research and Treatment of Cancer Quality of Life questionnaire; GHS/QoL, Global Health Score/quality of life; H&N35, Head and Neck 35; PF, physical functioning, PRO FAS, patient-reported outcomes full analysis set.

**Figure 2:**
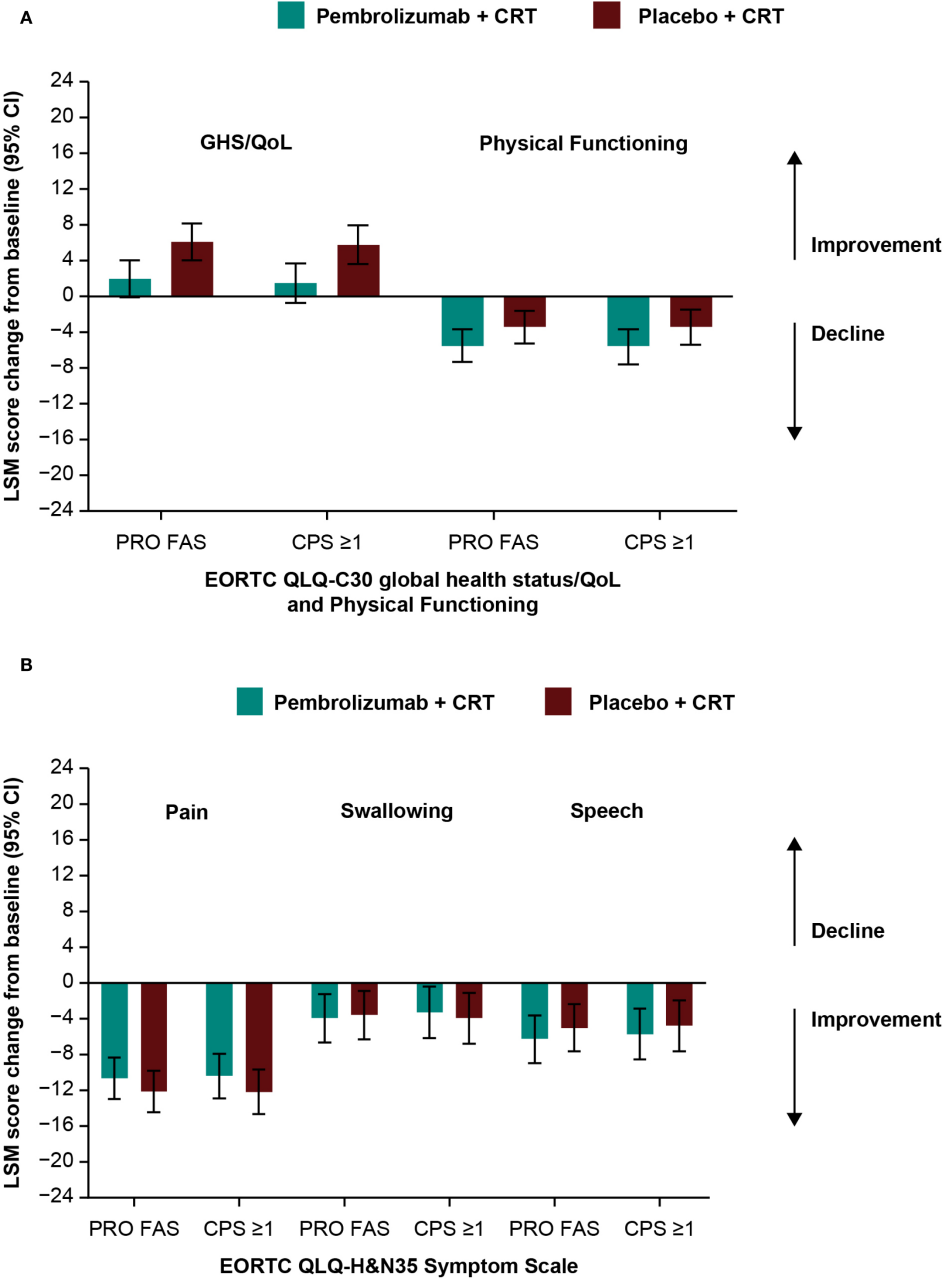
Difference in least squares mean from baseline to week 45 by PD-L1 CPS ≥1 in **(A)** EORTC QLQ-C30 GHS/QoL and PF and **(B)** EORTC QLQ-H&N35 pain, swallowing, and speech scores. C30, Core 30; CI, confidence interval; CPS, combined positive score; CRT, chemoradiotherapy; EORTC QLQ, European Organisation for Research and Treatment of Cancer Quality of Life questionnaire; GHS/QoL, Global Health Score/quality of life; H&N35, Head and Neck 35; LSM, least squares mean; PD-L1, programmed cell death ligand 1; PF, physical functioning, PRO FAS, patient-reported outcomes full analysis set.

**Figure 3:**
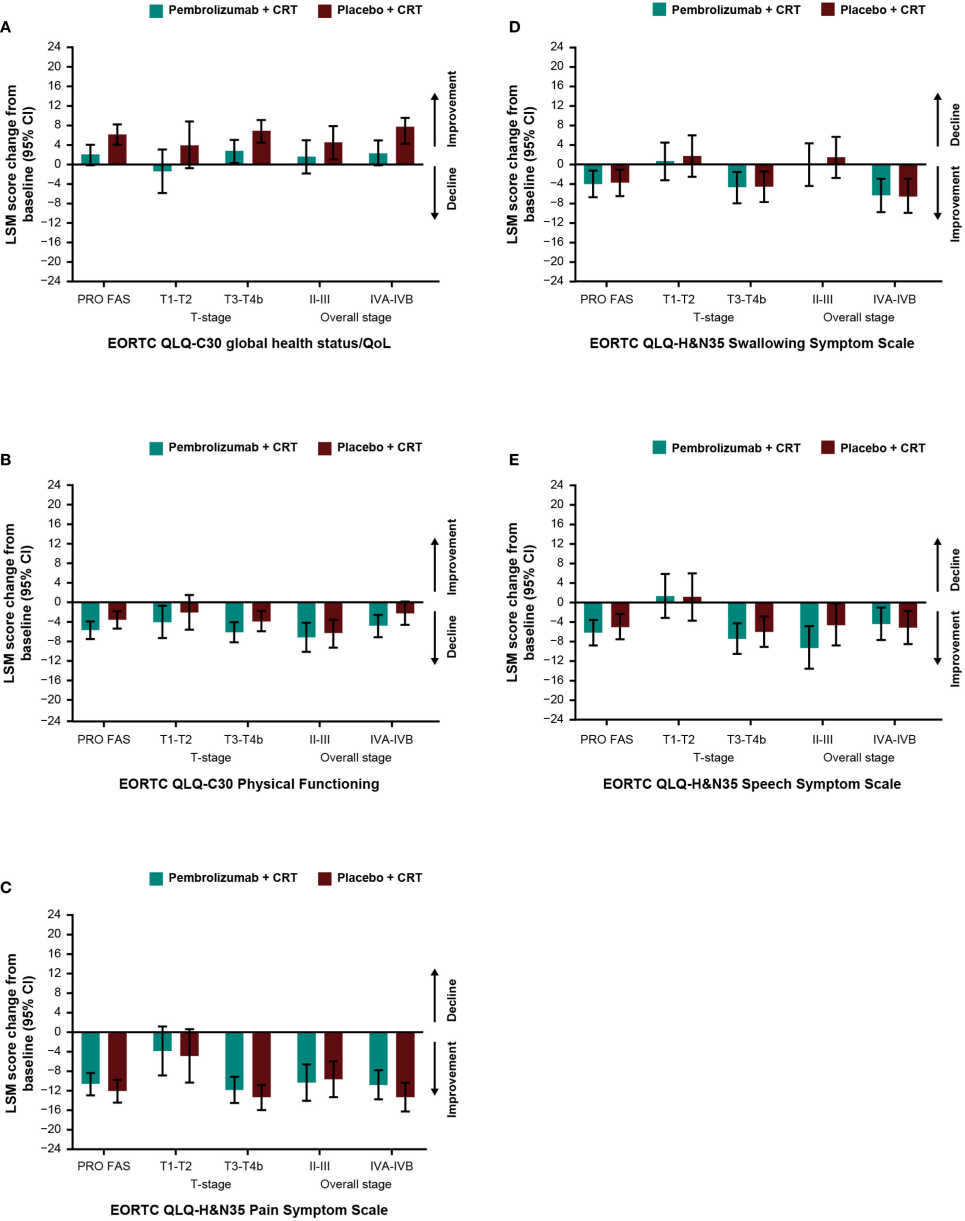
Difference in least squares mean from baseline to week 45 by cancer stage in **(A)** EORTC QLQ-C30 GHS/QoL **(B)** EORTC QLQ-C30 PF **(C)** EORTC QLQ-H&N35 pain **(D)** EORTC QLQ-H&N35 swallowing and **(E)** EORTC QLQ-H&N35 speech. C30, Core 30; CI, confidence interval; CRT, chemoradiotherapy; EORTC QLQ, European Organisation for Research and Treatment of Cancer Quality of Life questionnaire; GHS/QoL, Global Health Score/quality of life; H&N35, Head and Neck 35; LSM, least squares mean; PF, physical functioning, PRO FAS, patient-reported outcomes full analysis set.

Baseline scores for all PRO instruments were similar between treatment groups (**Table 1**). Baseline scores for EORTC QLQ-C30 and EORTC QLQ-H&N35 were similar between groups based on tumor location (**Supplementary Table S3**) and by T-stage and overall tumor staging (**Supplementary Table S4**). Treatment with pembrolizumab plus CRT resulted in LSM changes from baseline to week 45 ranging from -10.6 to +2.0 across EORTC QLQ-C30 and QLQ-H&N35 subscale scores (**Table 1**). An improvement from baseline in GHS/QoL scores was observed in the placebo plus CRT group compared with the pembrolizumab plus CRT group. Patient-reported outcomes remained generally stable over time in both treatment groups for measures of physical functioning or for disease-related symptom scores; however, an improvement from baseline in the EORTC QLQ-H&N35 pain symptom score was observed in both treatment groups (-10.6 [95% CI: -12.9 to -8.3] for participants treated with pembrolizumab plus CRT and -12.1 [95% CI: -14.4 to -9.8] for participants treated with placebo plus CRT). Results were generally similar to the total PRO population when analyzed by primary tumor site (**Figures 1A-E**) and by PD-L1 CPS ≥1 (**Figures 2A, B**), except for a decline in swallowing in participants in the pembrolizumab plus CRT group with hypopharynx as the primary tumor site. Results were also similar when analyzed by T-stage and by overall tumor staging, except for a decline in swallowing for T1-T2 stage and stage II-III in both treatment groups and a decline in speech for T1-T2 stage in both treatment groups (**Figures 3A-E**). There were no meaningful differences within or between treatment groups in EQ-5D VAS score.

**Table 1:** Mean change from baseline to week 45 in EORTC QLQ-C30, EORTC QLQ-H&N35, and EQ-5D subscale scores.

Assessment			Baseline		Week 45		Change from baseline to week 45		
			n^a^	Mean (SD)	n^a^	Mean (SD)	N^b^	LSM (95% CI)	Difference in LSM (95% CI)
EORTC QLQ	C30 GHS/QoL	Pembrolizumab + CRT	378	69.6 (20.7)	268	73.0 (18.1)	395	2.0 (-0.1 to 4.0)	-4.1 (-6.7 to -1.5)
		Placebo + CRT	382	67.8 (20.4)	266	76.3 (16.8)	397	6.1 (4.0 to 8.1)	
	C30 PF	Pembrolizumab + CRT	378	88.8 (15.8)	268	84.8 (17.0)	395	-5.5 (-7.3 to -3.7)	-2.1 (-4.5 to 0.4)
		Placebo + CRT	382	88.2 (16.0)	266	86.8 (15.1)	397	-3.5 (-5.3 to -1.7)	
	H&N35 pain	Pembrolizumab + CRT	378	25.8 (24.3)	268	15.0 (18.1)	395	-10.6 (-12.9 to -8.3)	1.44 (-1.3 to 4.2)
		Placebo + CRT	378	27.6 (25.6)	265	13.0 (16.1)	396	-12.1 (-14.4 to -9.8)	
	H&N35 swallowing	Pembrolizumab + CRT	378	22.5 (24.8)	268	18.3 (22.3)	395	-3.9 (-6.6 to -1.2)	-0.3 (-3.8 to 3.1)
		Placebo + CRT	378	25.1 (26.3)	265	17.7 (22.1)	396	-3.6 (-6.3 to -0.9)	
	H&N35 speech	Pembrolizumab + CRT	378	23.7 (27.2)	268	17.3 (21.6)	395	-6.2 (-8.9 to -3.6)	-1.3 (-4.6 to 2.1)
		Placebo + CRT	378	26.2 (27.7)	265	17.5 (22.2)	396	-5.0 (-7.6 to -2.3)	
EQ-5D	VAS	Pembrolizumab + CRT	384	75.6 (18.6)	268	78.8 (16.1)	395	3.5 (1.6 to 5.3)	-1.4 (-3.7 to 0.9)
		Placebo + CRT	386	72.1 (21.2)	266	79.9 (15.3)	397	4.9 (3.1 to 6.8)	

^a^n is the number of patients in each treatment group with non-missing assessments at the specific time point. ^b^N is the number of participants in the PRO analysis population in each treatment group.

C30, Core 30; CI, confidence interval; CRT, chemoradiotherapy; EORTC QLQ, European Organisation for Research and Treatment of Cancer Quality of Life questionnaire; EQ-5D, EuroQol-5 Dimensions; GHS/QoL, Global Health Score/quality of life; H&N35, Head and Neck 35; LSM, least squares mean; PF, physical functioning; PRO, patient-reported outcome; SD, standard deviation; VAS, visual analog scale.

**Figure 4:**
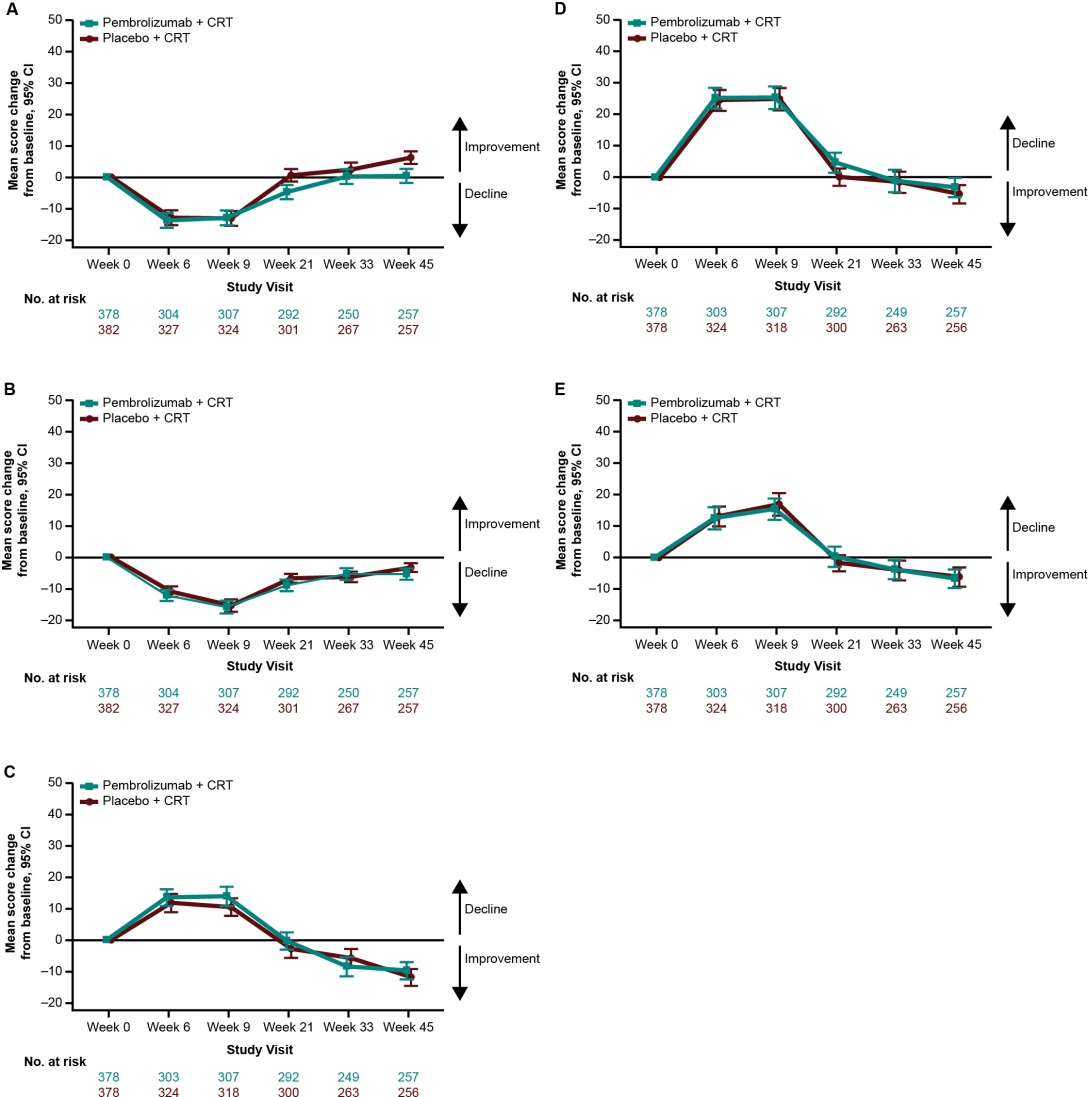
Empirical mean change from baseline to week 45 in **(A)** EORTC QLQ-C30 GHS/QoL, **(B)** EORTC QLQ-C30 PF, **(C)** EORTC QLQ-H&N35 pain, **(D)** EORTC QLQ-H&N35 swallowing, and **(E)** EORTC QLQ-H&N35 speech scores. C30, Core 30; CI, confidence interval; CRT, chemoradiotherapy; EORTC QLQ, European Organisation for Research and Treatment of Cancer Quality of Life questionnaire; GHS/QoL, Global Health Score/quality of life; H&N35, Head and Neck 35; PF, physical functioning.

**Figure 5:**
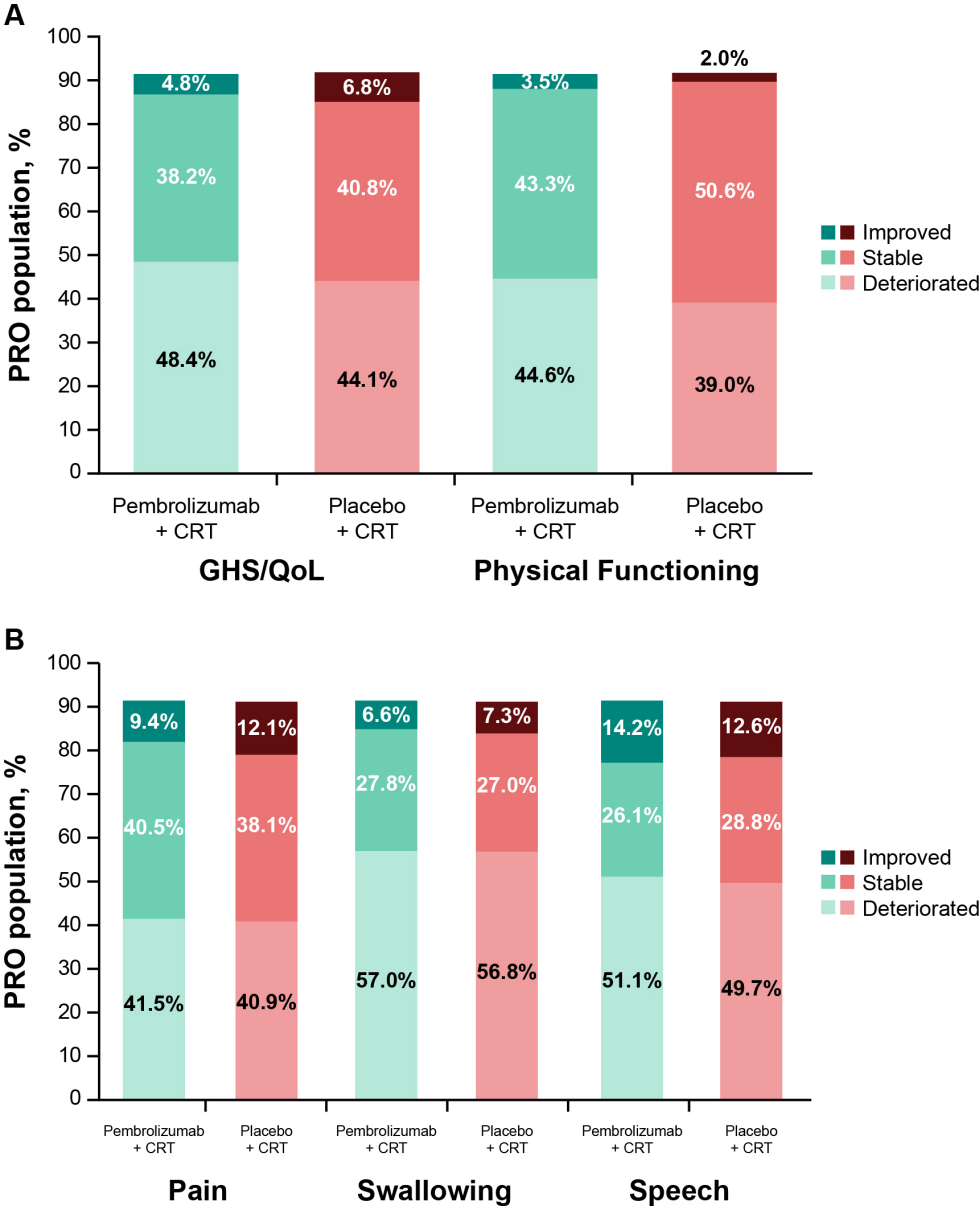
Proportion of participants with improved, stable, or deteriorated scores from baseline to week 45 in **(A)** EORTC QLQ-C30 GHS/QoL and PF, and **(B)** EORTC QLQ-H&N35 pain, swallowing, and speech. C30, Core 30; CRT, chemoradiotherapy; EORTC QLQ, European Organisation for Research and Treatment of Cancer Quality of Life questionnaire; GHS/QoL, Global Health Score/quality of life; H&N35, Head and Neck 35; PF, physical functioning; PRO, patient-reported outcome.

Empirical mean change from baseline to week 45 for EORTC QLQ-C30 GHS/QoL was generally stable, with a decline at the start of treatment and recovery to baseline by week 45 (**Figure 4A**). This pattern was evident for all assessed PRO scores (**Figures 4A–E**). There were no notable differences in empirical mean change from baseline to week 45 between treatment groups (**Figures 4A–E**). The proportion of participants with improvement, stability, or deterioration from baseline to week 45 was comparable between treatment groups for both EORTC QLQ-C30 (**Figure 5A**) and EORTC QLQ-H&N35 scores (**Figure 5B**).

## Discussion

In the KEYNOTE-412 study, participants with LA HNSCC treated with pembrolizumab plus CRT reported generally similar HRQoL outcomes as participants treated with placebo plus CRT. Results were generally similar by tumor site location, PD-L1 CPS ≥1, and cancer stage, and no major differences were observed in these subgroups. Declines were observed in both treatment groups for participants with T1–T2 stage (speech and swallowing) and stage II–III (swallowing) disease. There was also a decline in swallowing in the pembrolizumab plus CRT group with hypopharynx as the primary tumor site. Notably, an improvement in the EORTC QLQ-H&N35 pain symptom score was observed in both pembrolizumab plus CRT and placebo plus CRT groups. To the best of our knowledge, this is the first detailed report of PROs in participants with LA HNSCC treated with an immune checkpoint inhibitor.

Previous analyses of PROs in participants with HNSCC treated with pembrolizumab in phase 3 studies have shown comparable results. In KEYNOTE-040, participants with recurrent and/or metastatic (R/M) HNSCC treated with pembrolizumab had stable GHS/QoL scores (LSM [95% CI]: 0.38 [-3.00 to 3.78]) compared with participants treated with the standard of care (LSM [95% CI]: -5.86 [-9.68 to -2.04]) (16). Participants in both treatment groups had stable functioning and symptom scores. In KEYNOTE-048, participants with treatment-naïve R/M HNSCC were treated with pembrolizumab, pembrolizumab–chemotherapy, or cetuximab–chemotherapy; PRO scores remained stable, and GHS/QoL scores were similar between groups (17).

These results also largely align with the limited studies of PROs in participants with head and neck cancers treated with other therapies targeting the PD-(L)1 axis in the literature (18, 19). In the phase 3 CheckMate 141 and phase 4 VOLUME-PRO studies, participants with R/M HNSCC treated with nivolumab reported mostly stable symptoms and functioning, with few differences observed from baseline in EORTC QLQ-C30, QLQ-H&N35, and EQ-5D VAS scores (18, 20). Participants with R/M HNSCC participating in the phase 2 HAWK study who received durvalumab reported an improvement in EORTC QLQ-C30 GHS/QoL, physical functioning, and fatigue, as well as in EORTC QLQ-H&N35 mouth pain, swallowing, taste and smell, and speech symptom scores (21). An exploratory study evaluating PROs in participants with HNSCC starting treatment with immune checkpoint inhibitor monotherapy or combination therapy with cetuximab also found that QoL stabilized over time (19).

The participants in these prior studies had more advanced disease and had received previous treatment; given that these participants often face shorter survival times, changes in PROs from baseline were evaluated at earlier time points than the results presented in this analysis (week 15 in KEYNOTE-040 and KEYNOTE-048, weeks 9–15 in CheckMate 141, weeks 6–8 in VOLUME-PRO, and weeks 16–24 in HAWK) (17, 18, 20–22). This highlights the value of the extended follow-up period in this study, which can yield insights into the longer-term effect of treatment on the QoL of patients with head and neck cancer.

A common challenge in HRQoL studies is the decrease over time in the number of participants completing PRO assessments, which is a limitation of this analysis. Some subgroups had relatively small numbers of participants and KEYNOTE-412 was not powered to determine the statistical significance of PROs and lacked multiplicity control, so the results should be interpreted with caution.

In conclusion, PROs were similar between treatment with pembrolizumab plus CRT and placebo plus CRT in the first-line setting for participants with LA HNSCC, suggesting that the addition of pembrolizumab to CRT did not meaningfully impact HRQoL.

## Data Availability

The datasets presented in this article are not readily available because Merck Sharp & Dohme LLC, a subsidiary of Merck & Co., Inc., Rahway, NJ, USA (MSD) is committed to providing qualified scientific researchers access to anonymized data and clinical study reports from the company’s clinical trials for the purpose of conducting legitimate scientific research. MSD is also obligated to protect the rights and privacy of trial participants and, as such, has a procedure in place for evaluating and fulfilling requests for sharing company clinical trial data with qualified external scientific researchers. The MSD data sharing website (available at: https://externaldatasharing-msd.com) outlines the process and requirements for submitting a data request. Applications will be promptly assessed for completeness and policy compliance. Feasible requests will be reviewed by a committee of MSD subject matter experts to assess the scientific validity of the request and the qualifications of the requestors. In line with data privacy legislation, submitters of approved requests must enter into a standard data-sharing agreement with MSD before data access is granted. Data will be made available for request after product approval in the United States and European Union or after product development is discontinued. There are circumstances that may prevent MSD from sharing requested data, including country or region-specific regulations. If the request is declined, it will be communicated to the investigator. Access to genetic or exploratory biomarker data requires a detailed, hypothesis-driven statistical analysis plan that is collaboratively developed by the requestor and MSD subject matter experts; after approval of the statistical analysis plan and execution of a data-sharing agreement, MSD will either perform the proposed analyses and share the results with the requestor or will construct biomarker covariates and add them to a file with clinical data that is uploaded to an analysis portal so that the requestor can perform the proposed analyses. Requests to access the datasets should be directed to https://externaldatasharing-msd.com).
