# Supplementary material for: Health-related quality of life outcomes from KEYNOTE-412: chemoradiotherapy with or without pembrolizumab in participants with head and neck squamous cell carcinoma
Source: Front Oncol. 2025 Oct 6;15:1645509. doi: 10.3389/fonc.2025.1645509 (PMC12535876; doi:10.3389/fonc.2025.1645509)
Supplement: Supplementary file 1 [file DataSheet1.docx]

**Supplement**

**Supplementary Table 1.** Baseline characteristics of the ITT population in KEYNOTE-412.

| **Characteristic** | **Pembrolizumab + CRT (n=402)** | **Placebo + CRT (n=402)** |
| --- | --- | --- |
| Age, years, median (range) | 59 (27–81) | 59 (36–79) |
| ≥65 years, n (%) | 109 (27.1) | 96 (23.9) |
| Sex, n (%) |  |  |
| Male | 331 (82.3) | 329 (81.8) |
| Female | 71 (17.7_ | 73 (18.2) |
| Race/ethnicity, n (%) |  |  |
| American Indian or Alaska Native | 2 (0.5) | 0 |
| Asian | 54 (13.4) | 48 (11.9) |
| Black or African American | 10 (2.5) | 11 (2.7) |
| Multiple | 19 (4.7) | 28 (7.0) |
| White | 311 (77.4) | 311 (77.4) |
| Missing | 6 (1.5) | 4 (1.0) |
| Region, n (%) |  |  |
| North America | 43 (10.7) | 43 (10.7) |
| Western Europe | 198 (49.3) | 182 (45.3) |
| Rest of the world | 161 (40.0) | 177 (44.0) |
| PD-L1 CPS,^a^ n (%) |  |  |
| <1 | 46 (11.4) | 36 (9.0) |
| ≥1 | 339 (84.3) | 346 (86.1) |
| ≥20 | 146 (36.3) | 145 (36.1) |
| Missing | 17 (4.2) | 20 (5.0) |
| ECPG PS, n (%) |  |  |
| 0 | 265 (6.6) | 251 (6.2) |
| 1 | 137 (34.1) | 151 (37.6) |
| Primary tumor site, n (%) |  |  |
| Oropharynx | 200 (49.8) | 204 (50.7) |
| Larynx | 92 (22.9) | 86 (21.4) |
| Hypopharynx | 71 (17.7) | 73 (18.2) |
| Oral cavity | 39 (9.7) | 39 (9.7) |
| HPV status, n (%) |  |  |
| Negative | 293 (72.9) | 298 (74.1) |
| Positive | 109 (27.1) | 104 (25.9) |
| Radiotherapy regimen, n (%) |  |  |
| Standard fractionation | 391 (97.3) | 393 (97.8) |
| Accelerated fractionation | 11 (2.7) | 9 (2.2) |
| Overall stage,^b^ n (%) |  |  |
| II–III^c^ | 140 (34.8) | 137 (34.1) |
| IVA–IVB | 262 (65.2) | 265 (65.9) |
| Tumor stage,^b,d^ n (%) |  |  |
| T1–T2 | 67 (16.7) | 54 (13.4) |
| T3–T4b | 335 (83.3) | 347 (86.3) |
| Nodal stage,^b,e^ n (%) |  |  |
| N0–N1 | 122 (30.3) | 134 (33.3) |
| N2a–c | 225 (56.0) | 222 (55.2) |
| N3a–b | 54 (13.4) | 46 (11.4) |
| Baseline tumor burden |  |  |
| Participants with data, n | 383 | 389 |
| Median (range), mm | 50 (10–173) | 50 (12–264) |
| Smoking status, n (%) |  |  |
| Former or current smoker | 346 (86.1) | 346 (86.1) |
| Never | 56 (13.9) | 54 (13.4) |
| Missing | 0 | 2 (0.5) |

^a^PD-L1 expression was determined using PD-L1 IHC 22C3 pharmDx (Agilent, Santa Carla, CA, USA). CPS was calculated as the total number of PD-L1–staining cells (tumor cells, lymphocytes, and macrophages) divided by the total number of viable tumor cells, multiplied by 100. ^b^Tumors were staged according to the American Joint Committee on Cancer staging manual (7th edition for participants enrolled before 1 January 2018; 8th edition for participants enrolled on or after 1 January 2018). ^c^One participant in the placebo + CRT group had stage II disease at baseline and was enrolled in error. ^d^One participant in the placebo + CRT group was staged as TX. ^e^One participant in the pembrolizumab + CRT group was staged as NX.

CPS, combined positive score; CRT, chemoradiotherapy; ECOG PS, Eastern Cooperative Oncology Group performance status; HPV, human papilloma virus; ITT, intention-to-treat; PS, performance score; PD-L1, programmed cell death ligand 1.

**Supplementary Table 2.** Completion^a^ and compliance^b^ rates for PRO assessment tools.

| **Treatment visit, % (n/N)** | **EORTC QLQ-C30** | | **EORTC QLQ-H&N35** | | **EQ-5D** | |
| --- | --- | --- | --- | --- | --- | --- |
|  | **Pembrolizumab + CRT (n=395)** | **Placebo + CRT (n=397)** | **Pembrolizumab + CRT (n=395)** | **Placebo + CRT (n=396)** | **Pembrolizumab + CRT (n=395)** | **Placebo + CRT (n=397)** |
| Baseline |  |  |  |  |  |  |
| Completion | 95.7 (378/395) | 96.2 (382/397) | 95.7 (378/395) | 95.5 (378/396) | 97.2 (384/395) | 97.2 (386/397) |
| Compliance | 99.5 (378/380) | 100.0 (382/382) | 99.5 (378/380) | 100.0 (378/378) | 97.2 (384/395) | 97.2 (386/397) |
| Week 6 |  |  |  |  |  |  |
| Completion | 80.5 (318/395) | 85.1 (338/397) | 80.3 (317/395) | 85.1 (337/396) | 81.5 (322/395) | 85.9 (341/397) |
| Compliance | 91.9 (318/346) | 96.8 (338/349) | 91.9 (317/345) | 96.8 (337/348) | 81.7 (322/394) | 87.2 (341/391) |
| Week 9 |  |  |  |  |  |  |
| Completion | 80.5 (318/395) | 84.4 (335/397) | 80.5 (318/395) | 83.3 (330/396) | 81.0 (320/395) | 84.6 (336/397) |
| Compliance | 98.8 (318/322) | 98.5 (335/340) | 98.8 (318/322) | 98.5 (330/335) | 84.2 (320/380) | 90.6 (336/371) |
| Week 21 |  |  |  |  |  |  |
| Completion | 77.0 (304/395) | 77.8 (309/397) | 77.0 (304/395) | 78.0 (309/396) | 77.7 (307/395) | 78.6 (312/397) |
| Compliance | 95.3 (304/319) | 94.5 (309/327) | 95.3 (304/319) | 94.5 (309/327) | 85.3 (307/360) | 85.5 (312/365) |
| Week 33 |  |  |  |  |  |  |
| Completion | 66.1 (261/395) | 69.3 (275/397) | 65.8 (260/395) | 68.7 (272/396) | 67.1 (265/395) | 70.3 (279/397) |
| Compliance | 91.3 (261/286) | 95.5 (275/288) | 91.2 (260/285) | 95.4 (272/285) | 82.0 (265/323) | 86.6 (279/322) |
| Week 45 |  |  |  |  |  |  |
| Completion | 67.8 (268/395) | 67.0 (266/397) | 67.8 (268/395) | 66.9 (265/396) | 67.8 (268/395) | 67.0 (266/397) |
| Compliance | 97.1 (268/276) | 97.4 (266/273) | 97.1 (268/276) | 97.4 (265/272) | 90.5 (268/296) | 93.3 (266/285) |
| Year 2, Quarter 1 |  |  |  |  |  |  |
| Completion | 57.0 (225/395) | 51.1 (203/397) | 57.0 (225/395) | 51.0 (202/396) | 57.0 (225/395) | 50.9 (202/397) |
| Compliance | 100.0 (225/225) | 100.0 (203/203) | 100.0 (225/225) | 100.0 (202/202) | 86.2 (225/261) | 85.2 (202/237) |
| Year 2, Quarter 2 |  |  |  |  |  |  |
| Completion | 42.8 (169/395) | 39.5 (157/397) | 42.5 (168/395) | 39.6 (157/396) | 42.8 (169/395) | 39.5 (157/397) |
| Compliance | 100.0 (169/169) | 100.0 (157/157) | 100.0 (168/168) | 100.0 (157/157) | 79.3 (169/213) | 84.9 (157/185) |
| Year 2, Quarter 3 |  |  |  |  |  |  |
| Completion | 38.7 (153/395) | 37.3 (148/397) | 38.5 (152/395) | 37.1 (147/396) | 38.5 (152/395) | 37.5 (149/397) |
| Compliance | 100.0 (153/153) | 100.0 (148/148) | 100.0 (152/152) | 100.0 (147/147) | 75.6 (152/201) | 82.8 (149/180) |
| Year 2, Quarter 4 |  |  |  |  |  |  |
| Completion | 32.9 (130/395) | 27.2 (108/397) | 32.9 (130/395) | 27.0 (107/396) | 32.9 (130/395) | 27.7 (110/397) |
| Compliance | 100.0 (130/130) | 100.0 (108/108) | 100.0 (130/130) | 100.0 (107/107) | 78.3 (130/166) | 78.6 (110/140) |
| End of Year 3 |  |  |  |  |  |  |
| Completion | 40.5 (160/395) | 38.5 (153/397) | 40.5 (160/395) | 39.1 (155/396) | 40.5 (160/395) | 39.0 (155/397) |
| Compliance | 100.0 (160/160) | 100.0 (153/153) | 100.0 (160/160) | 100.0 (155/155) | 76.2 (160/210) | 81.2 (155/191) |

^a^Completion is defined as the proportion of participants who completed ≥1 questionnaire at each time point among those in the PRO analysis population. ^b^Compliance is defined as the proportion of participants who completed ≥1 questionnaire at each time point among those who are expected to complete the instruments at that time point, excluding those missing by design (such as death, discontinuation, and translations not available).

C30, Core 30; CRT, chemoradiotherapy; EORTC QLQ, European Organisation for Research and Treatment of Cancer Quality of Life questionnaire; EQ-5D, EuroQol-5 Dimensions; H&N35, Head and Neck 35; PRO, patient-reported outcome.

**Supplementary Table 3.** Baseline EORTC QLQ-C30 and EORTC QLQ-H&N35 subscale scores by primary tumor site and PD-L1 CPS ≥1

| **Assessment** | | **PRO FAS** | | **Larynx** | | **Hypopharynx** | | **Oropharynx** | | **Oral Cavity** | | **PD-L1 CPS ≥1** | |
| --- | --- | --- | --- | --- | --- | --- | --- | --- | --- | --- | --- | --- | --- |
|  |  | **n^a^** | **Mean (SD)** | **n^a^** | **Mean (SD)** | **n^a^** | **Mean (SD)** | **n^a^** | **Mean (SD)** | **n^a^** | **Mean (SD)** | **n^a^** | **Mean (SD)** |
| GHS/QoL | Pembrolizumab + CRT | 378 | 69.6 (20.7) | 84 | 73.0 (22.2) | 66 | 67.8 (20.0) | 192 | 69.6 (20.1) | 36 | 64.8 (20.5) | 320 | 69.3 (20.6) |
|  | Placebo + CRT | 382 | 67.8 (20.4) | 84 | 69.4 (18.0) | 68 | 65.0 (21.9) | 192 | 69.0 (20.7) | 38 | 63.8 (21.1) | 329 | 68.2 (20.5) |
| PF | Pembrolizumab + CRT | 378 | 88.8 (15.8) | 84 | 88.3 (17.0) | 66 | 90.5 (13.8) | 192 | 89.4 (14.0) | 36 | 83.3 (23.1) | 320 | 88.7 (15.9) |
|  | Placebo + CRT | 382 | 88.2 (16.0) | 84 | 89.1 (13.5) | 68 | 85.3 (18.7) | 192 | 88.9 (15.5) | 38 | 87.9 (17.7) | 329 | 88.4 (15.7) |
| H&N35 pain | Pembrolizumab + CRT | 378 | 25.8 (24.3) | 84 | 13.8 (15.7) | 66 | 23.2 (20.8) | 192 | 28.9 (25.8) | 36 | 42.4 (25.2) | 320 | 26.9 (24.5) |
|  | Placebo + CRT | 378 | 27.6 (25.6) | 84 | 14.2 (14.6) | 67 | 22.3 (21.7) | 189 | 32.4 (27.6) | 38 | 41.9 (26.2) | 325 | 27.5 (25.4) |
| H&N35 swallowing | Pembrolizumab + CRT | 378 | 22.5 (24.8) | 84 | 16.8 (24.1) | 66 | 28.8 (23.9) | 192 | 21.7 (23.8) | 36 | 28.7 (29.4) | 320 | 22.9 (24.7) |
|  | Placebo + CRT | 378 | 25.1 (26.3) | 84 | 18.1 (22.8) | 67 | 30.7 (27.9) | 189 | 25.6 (26.6) | 38 | 28.3 (26.5) | 325 | 24.5 (25.7) |
| H&N35 speech | Pembrolizumab + CRT | 378 | 23.7 (27.2) | 84 | 41.0 (32.0) | 66 | 23.9 (24.2) | 192 | 15.3 (21.4) | 36 | 28.1 (28.3) | 320 | 23.8 (26.6) |
|  | Placebo + CRT | 378 | 26.2 (27.7) | 84 | 39.3 (30.8) | 67 | 23.6 (27.7) | 189 | 20.9 (24.7) | 38 | 28.7 (25.4) | 325 | 24.9 (26.2) |

^a^n is the number of participants in each treatment group with non-missing assessments at the specific time point.

C30, Core 30; CI, confidence interval; CRT, chemoradiotherapy; CPS, combined positive score; EORTC QLQ, European Organisation for Research and Treatment of Cancer Quality of Life questionnaire; GHS/QoL, Global Health Score/quality of life; H&N35, Head and Neck 35; PD-L1, programmed cell death ligand 1; PF, physical functioning; PRO FAS, patient-reported outcome full analysis set; SD, standard deviation.

**Supplementary Table 4.** Baseline EORTC QLQ-C30 and EORTC QLQ-H&N35 subscale scores by T-stage and overall staging

| **Assessment** | | **PRO FAS** | | **T1-T2** | | **T3-T4b** | | **II-III^a^** | | **IVa-IVb** | |
| --- | --- | --- | --- | --- | --- | --- | --- | --- | --- | --- | --- |
|  |  | **n^b^** | **Mean (SD)** | **n^b^** | **Mean (SD)** | **n^b^** | **Mean (SD)** | **n^b^** | **Mean (SD)** | **n^b^** | **Mean (SD)** |
| GHS/QoL | Pembrolizumab + CRT | 378 | 69.6 (20.7) | 64 | 74.4 (18.4) | 314 | 68.6 (21.0) | 133 | 72.6 (20.9) | 245 | 68.0 (20.4) |
|  | Placebo + CRT | 382 | 67.8 (20.4) | 53 | 72.2 (20.7) | 328 | 67.1 (20.4) | 133 | 70.9 (18.8) | 249 | 66.2 (21.1) |
| PF | Pembrolizumab + CRT | 378 | 88.8 (15.8) | 64 | 94.9 (9.5) | 314 | 87.5 (16.5) | 133 | 90.2 (14.1) | 245 | 88.0 (16.6) |
|  | Placebo + CRT | 382 | 88.2 (16.0) | 53 | 89.6 (16.7) | 328 | 87.9 (15.9) | 133 | 91.1 (11.9) | 249 | 86.6 (17.6) |
| H&N35 pain | Pembrolizumab + CRT | 378 | 25.8 (24.3) | 64 | 16.5 (18.8) | 314 | 27.7 (24.9) | 133 | 25.8 (27.1) | 245 | 25.9 (22.6) |
|  | Placebo + CRT | 378 | 27.6 (25.6) | 51 | 16.8 (19.3) | 326 | 29.2 (26.0) | 132 | 26.6 (26.2) | 246 | 28.0 (25.3) |
| H&N35 swallowing | Pembrolizumab + CRT | 378 | 22.5 (24.8) | 64 | 7.4 (11.1) | 314 | 25.6 (25.6) | 133 | 18.3 (23.9) | 245 | 24.8 (25.0) |
|  | Placebo + CRT | 378 | 25.1 (26.3) | 51 | 14.2 (17.9) | 326 | 26.9 (27.0) | 132 | 17.9 (23.7) | 246 | 28.9 (26.8) |
| H&N35 speech | Pembrolizumab + CRT | 378 | 23.7 (27.2) | 64 | 9.0 (10.8) | 314 | 26.7 (28.5) | 133 | 23.1 (26.9) | 245 | 24.0 (27.3) |
|  | Placebo + CRT | 378 | 26.2 (27.7) | 51 | 15.7 (24.1) | 326 | 28.0 (27.9) | 132 | 23.9 (27.6) | 246 | 27.5 (27.7) |

^a^1 participant in the placebo plus CRT group was enrolled with stage II disease at baseline in error.
^b^n is the number of participants in each treatment group with non-missing assessments at the specific time point.

C30, Core 30; CI, confidence interval; CRT, chemoradiotherapy; CPS, combined positive score; EORTC QLQ, European Organisation for Research and Treatment of Cancer Quality of Life questionnaire; GHS/QoL, Global Health Score/quality of life; H&N35, Head and Neck 35; PF, physical functioning; PRO FAS, patient-reported outcome full analysis set; SD, standard deviation.
